# Coyotes as Reservoirs for *Onchocerca lupi*, United States, 2015–2018

**DOI:** 10.3201/eid2612.190136

**Published:** 2020-12

**Authors:** Chandler C. Roe, Hayley Yaglom, April Howard, Jennifer Urbanz, Guilherme G. Verocai, Lela Andrews, Veronica Harrison, Riley Barnes, Ted Lyons, Jolene R. Bowers, David M. Engelthaler

**Affiliations:** Translational Genomics Research Institute, Flagstaff, Arizona, USA (C.C. Roe, H. Yaglom, V. Harrison, R. Barnes, T. Lyons, J.R. Bowers, D.M. Engelthaler);; Northern Arizona University, Flagstaff (C.C. Roe, L. Andrews);; Arizona Department of Health Services, Phoenix, Arizona, USA (H. Yaglom);; Arizona Game and Fish Department, Phoenix (A. Howard);; Eye Care for Animals, Scottsdale, Arizona, USA (J. Urbanz);; Texas A&M University, College Station, Texas, USA (G.G. Verocai);; University of Georgia, Athens, Georgia, USA (G.G. Verocai)

**Keywords:** coyotes, Onchocerca lupi, amplicon sequencing, phylogenetics, nematodes, parasites, zoonoses, United States, Canis latrans, Arizona, New Mexico, Nevada, onchocerciasis

## Abstract

The *Onchocerca lupi* nematode infects dogs, cats, and humans, but whether it can be spread by coyotes has been unknown. We conducted surveillance for *O. lupi* nematode infection in coyotes in the southwestern United States. We identified multiple coyote populations in Arizona and New Mexico as probable reservoirs for this species.

*Onchocerca lupi* is a species of zoonotic, filarial nematode that causes onchocerciasis in dogs, cats, and humans. It was first described in 1967 in Georgia, then part of the USSR, in the periocular tissues of a wolf (*Canis lupus lupus*) (*1*) and has been reported in dogs (*C. lupus domesticus*). Since 2013, increased detection of *O. lupi* infections in dogs and humans in the United States and Europe has renewed interest in this parasite, its geographic distribution, and the range of its natural hosts (*2*).

The geographic distribution and prevalence of the *O. lupi* nematode in the United States is unknown. US veterinarians are not required to report *O. lupi* infections in canines, making it difficult to identify the parasite’s geographic distribution. The first documented case of *O. lupi* infection in the United States affected a dog in California in 1991 (*3*); since then, *O.* *lupi* infections have been reported in dogs, cats, and humans in Arizona, California, Colorado, New Mexico, Texas, and Utah (*2*,*4*,*5*,*6*). This parasitic nematode is now endemic in domesticated canines in the southwestern United States (*7*). Reports of *O. lupi* infection in Canada (Alberta and Prince Edward Island) (*7*) associated with dog importation from the southwestern United States and travel of US companion animals suggest an anthropogenic spread of the *O. lupi* nematode. Whether wild canids, including coyotes (*C. latrans*), might be reservoirs for the *O. lupi* nematode is unknown.

Because of the growing number of *O. lupi* infections in canines and humans, public health officials must understand the prevalence and distribution of this parasite in wildlife. Toward that goal, we investigated coyote populations in Arizona, New Mexico, and Nevada as potential primary hosts and natural reservoirs for the *O. lupi* nematode. 

## The Study

From December 2015 through July 2018, we collected skin tissue samples from coyotes harvested for predation management and from a hunt in Arizona, New Mexico, and Nevada conducted by the Arizona Game and Fish Department. We did not euthanize any coyotes for the specific purpose of this study. Skin tissue from the interocular frontal area of the animal’s head was removed and stored in 80% ethanol until we extracted the DNA. We screened 707 DNA sequences for an *O. lupi* cytochrome c oxidase (COI) gene (*8*) using SYBR Green–real-time PCR on a QuantStudio 7 Flex Real Time PCR System (Thermo Fisher Scientific, https://www.thermofisher.com). We used DNA from an adult worm from an infected dog in northern Arizona as a positive control (GenBank accession no. MT878136). We included a no-template control in every real-time PCR reaction plate. We compared the product’s melting curve to the positive control using a dissociation curve. We prepared every sample that had a melting curve resembling that of the positive control for amplicon sequencing using neat DNA and Illumina (https://www.illumina.com) technologies.

**Figure 1:**
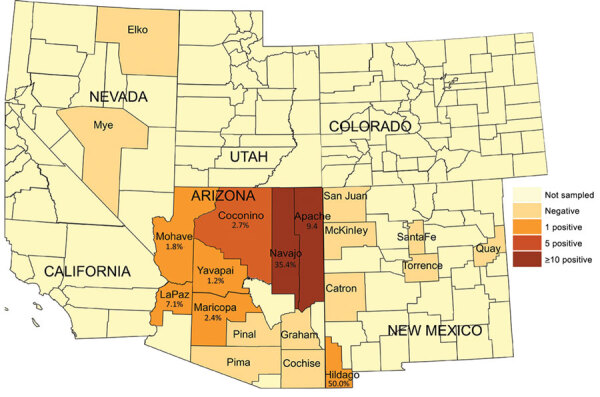
Number of *Onchocerca lupi* nematode–positive coyotes collected, southwestern United States, 2015–2018. Positivity rates are provided for each county with *O. lupi*–positive coyotes.

Thirty-seven (5.2%) samples from 8 counties in Arizona and New Mexico (Table) had sequences that aligned with the reference gene. Of these samples, 36 were from Arizona and 1 was from Hildago County, New Mexico. In Arizona, the highest prevalences of *O. lupi* infection were in Navajo County (17 [35.4%] positive coyotes) and Apache County (10 [9.4%]) (Figure 1). Coconino County had the third highest number (5) of coyotes that tested positive for the *O. lupi* nematode but a lower positivity rate (2.7%) than other counties. For example, in Hidalgo County we sampled only 2 coyotes, 1 of which tested positive.

**Table:** Coyotes tested for *Onchocerca lupi* nematodes, United States, 2015–2018

Location	No. samples	No. (%) positive samples	Coyote sex			Coyote age group	
			M	F		Adult	Youth
Arizona							
Coconino	189	5 (2.7)	97	92		177	12
Apache	106	10 (9.4)	56	50		88	18
Yavapai	86	1 (1.2)	49	37		78	8
Cochise	75	0	44	31		66	9
Mohave*	56	1 (1.8)	29	26		53	3
Navajo*	48	17 (35.4)	31	16		45	3
Graham	48	0	24	24		46	2
Maricopa	42	1 (2.4)	19	23		32	10
La Paz	14	1 (7.1)	5	9		12	2
Pima	6	0	2	4		6	0
Pinal	3	0	1	2		3	0
Yuma	1	0	1	0		1	0
Unknown†	1	0	0	1		1	0
Unknown†	1	0	0	1		1	0
New Mexico							
Catron	7	0	5	2		6	1
McKinley	1	0	0	1		1	0
San Juan	4	0	1	3		4	0
Torrence	4	0	3	1		3	1
Tucamari	3	0	1	2		3	0
Quay	3	0	3	0		3	0
Hildago	2	1 (50.0)	1	1		2	0
Zuni	1	0	1	0		1	0
Nevada							
Elko	4	0	3	1		4	0
Nye	2	0	1	1		2	0

*Mohave and Navajo Counties each had 1 sample for which host sex was not recorded. †2 samples from Arizona did not have a recorded county.

**Figure 2:**
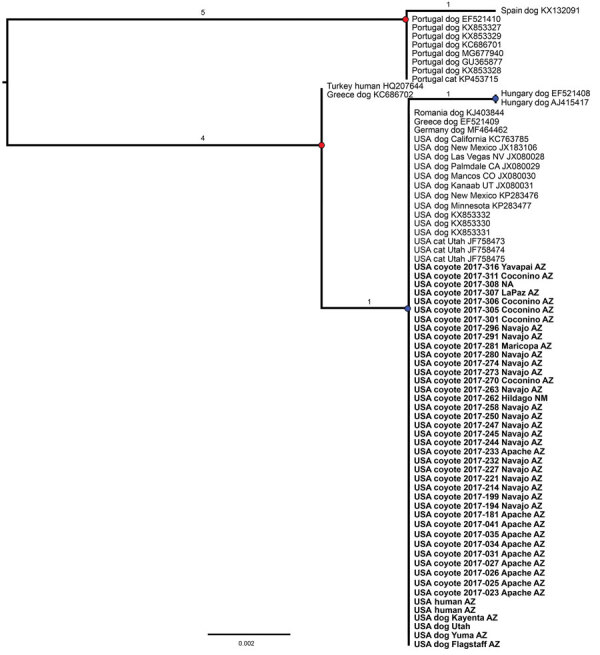
Rooted maximum-likelihood phylogenetic tree based on the cytochrome oxidase c gene sequence from 73 *Onchocerca lupi* nematode samples, including 43 newly obtained samples from 37 coyotes, 4 dogs, and 2 humans, southwestern United States, 2015–2018. This analysis covers 432 bases. Branch lengths indicate the number of single-nucleotide polymorphisms; red dots indicate bootstrap values >99; blue dots indicate bootstrap values <65. Countries of collection, host species, and year of collection are indicated. Newly sequenced specimens are in bold. Scale bar indicates number of nucleotide substitutions per site.

We produced a phylogenetic tree of our 43 COI sequences (including 4 that were isolated from infected dogs and 2 from humans [GenBank accession nos. MT878134–9]) in addition to 30 *O. lupi* COI genes on GenBank spanning 432 total bases containing 12 single-nucleotide polymorphisms (SNPs) (Figure 2) using IQTREE version 1.6.9 (*9*) software with 1,000 bootstrap replicates. Examining only this region of the COI gene, we determined the US samples (from dogs, cats, coyotes, and humans) clustered within a single clade with dog samples from Germany, Romania, and Greece. Within this clade, we detected no SNP differences. This clade was separated from a sample from Hungary by 1 SNP and from a single clade containing a human isolate from Turkey and a dog sample from Greece by 1 SNP.

## Conclusions

*O. lupi* infection has been reported mainly in domestic dogs and cats in the southwestern United States (*2**,**4*). However, international transportation (purchasing, adopting, and exporting) of dogs from that area has introduced this parasite into environments to which it is not endemic (*7*,*10*). We hypothesize coyotes are reservoirs for the *O. lupi* nematode and could spread this parasite throughout the southwestern United States. 

We consider the probable importance of coyotes as natural reservoirs and dispersal agents. In the United States, the average home territory covered by a resident coyote population (either a pack or lone coyote) is 5–41 km^2^, whereas solitary transient coyote territories are up to 155 km^2^ (*11*,*12*). The large geographic range and widespread occurrence of not only coyotes, but also the putative black fly vector (Diptera: *Simuliidae*) (*13*), might facilitate the spread and establishment of the *O. lupi* nematode in the southwestern United States. Furthermore, many North American wild canids, such as wolves and foxes, have never been assessed for the *O. lupi* nematode but also should be considered as potential reservoirs. Although the *O. lupi* nematode is only endemic to the southwestern United States, without appropriate surveillance and mitigation strategies it might spread across the United States and into Canada and Mexico. We are not aware of any reports of *O. lupi* nematodes in Mexico; however, we identified a coyote that tested positive for *O. lupi* infection in Hildago County, which borders Mexico. Increasing surveillance in nearby counties upon identification of *O. lupi* nematode–positive coyotes would be prudent. Furthermore, the overlap of rural human residences with coyote and black fly populations probably increases the risk for human exposure.

In summary, canine onchocerciasis is an ongoing emerging infectious threat to wildlife, companion animals, and humans. The expanding range to which the *O. lupi* nematode is endemic, coupled with increased incidence of onchocerciasis in humans and canines in the southwestern United States, reinforces the need to understand, respond to, and potentially mitigate this threat. This understanding will enable the development of surveillance and mitigation strategies, determine the risk of spread to nonendemic regions, and identify human populations at high risk of infection. 

## References

[1] Rodonaja TE. A new species of nematode, *Onchocerca lupi* n. sp., from *Canis lupus cubanensis.* Soobshchenyia Akad Nuak Gruz Ssr. 1967;45:715–9.

[2] Cantey PT, Weeks J, Edwards M, Rao S, Ostovar GA, Dehority W, et al. The emergence of zoonotic *Onchocerca lupi* infection in the United States—a case-series. Clin Infect Dis. 2016;62:778–83. 10.1093/cid/civ983 26611778 PMC4809994

[3] Orihel TC, Ash LR, Holshuh HJ, Santenelli S. Onchocerciasis in a California dog. Am J Trop Med Hyg. 1991;44:513–7. 10.4269/ajtmh.1991.44.513 2063954

[4] McLean NJ, Newkirk K, Adema CM. Canine ocular onchocerciasis: a retrospective review of the diagnosis, treatment, and outcome of 16 cases in New Mexico (2011-2015). Vet Ophthalmol. 2017;20:349–56. 10.1111/vop.12433 27624855 PMC5348283

[5] Eberhard ML, Ostovar GA, Chundu K, Hobohm D, Feiz-Erfan I, Mathison BA, et al. Zoonotic *Onchocerca lupi* infection in a 22-month-old child in Arizona: first report in the United States and a review of the literature. Am J Trop Med Hyg. 2013;88:601–5. 10.4269/ajtmh.12-0733 23382171 PMC3592550

[6] Labelle AL, Maddox CW, Daniels JB, Lanka S, Eggett TE, Dubielzig RR, et al. Canine ocular onchocercosis in the United States is associated with *Onchocerca lupi.* Vet Parasitol. 2013;193:297–301. 10.1016/j.vetpar.2012.12.002 23276598

[7] Verocai GG, Conboy G, Lejeune M, Marron F, Hanna P, MacDonald E, et al. *Onchocerca lupi* nematodes in dogs exported from the United States into Canada. Emerg Infect Dis. 2016;22:1477–9. 10.3201/eid2208.151918 27434170 PMC4982182

[8] Hassan HK, Bolcen S, Kubofcik J, Nutman TB, Eberhard ML, Middleton K, et al. Isolation of *Onchocerca lupi* in dogs and black flies, California, USA. Emerg Infect Dis. 2015;21:789–96. 10.3201/eid2105.142011 25897954 PMC4412245

[9] Nguyen L-T, Schmidt HA, von Haeseler A, Minh BQ. IQ-TREE: a fast and effective stochastic algorithm for estimating maximum-likelihood phylogenies. Mol Biol Evol. 2015;32:268–74. 10.1093/molbev/msu300 25371430 PMC4271533

[10] Colella V, Lia RP, Di Paola G, Cortes H, Cardoso L, Otranto D. International dog travelling and risk for zoonotic *Onchocerca lupi.* Transbound Emerg Dis. 2018;65:1107–9. 10.1111/tbed.12842 29476600

[11] Gehrt SD, Anchor C, White LA. Home range and landscape use of coyotes in a metropolitan landscape: conflict or coexistence? J Mammal. 2009;90:1045–57. 10.1644/08-MAMM-A-277.1

[12] Howard VW, Delfrate GG. Home ranges and movements of coyotes in the northern Chihuahuan desert. 1991 [cited 2019 Jan 4]. https://digitalcommons.unl.edu/cgi/viewcontent.cgi?article=1014&context=gpwdcwp

[13] Adler PH, Currie DC, Wood M, Idema RM, Zettler LW. The black flies (Simuliidae) of North America. Ithaca (NY): Cornell University Publishing; 2004.

